# A New System of Organic Chemistry, Translated from the French of F. V. Raspail

**Published:** 1834-10-01

**Authors:** 


*Nouveau Système de Chimie Organique, fondé sur des Méthodes Nouvelles d'Observation;* par F. V. RASPAIL. *Accompagné de douze Planches gravées, dont six coloriées.*—Paris, 1833. 8vo. pp. 576 and 96.

*A New System of Organic Chemistry, translated from the French of* F. V. RASPAIL. *With Notes and Additions, by* WILLIAM HENDERSON, M.D., Lecturer on Materia Medica in the University of Aberdeen.—London, 1834. 8vo. pp. 602 and lxxvi. Plates.

M. RASPAIL is a man of great talents and unquenchable zeal, who has no more respect for the right divine of chemists than of kings, and who, in the course of the very curious work before us, deals out sundry hard knocks to people of note, with the hearty goodwill of a man who is perfectly in earnest. We give the dedication, because it is so very French, and so very original.

"A la mémoire d'un homme de bien, mon pauvre maître, l'Abbé Eysséric.

"A toi qui sus allier le prêtre de l'évangile avec l'homme de la science et de la civilisation! A toi qui, à Paris, aurais mérité de n'être d'aucune académie, et qui, dans mon village, ne voulus jamais t'élever au-dessus de la dignité d'instituteur des pauvres! A toi, philologue d'une immense érudition, qui te dévouas toute ta vie à faire épeler des lettres! A toi, prêtre, qui n'a jamais voulu vivre que du travail de tes mains!

"A toi l'hommage de ce livre!

"Puisse cette consécration pieuse le rendre aussi utile à la science que l'ont toujours été tes exemples et tes leçons à la cause de l'humanité!

"Adieu. RASPAIL.

"*Maison d'Arrêt de Versailles;* 20 *Mars,* 1833."

Many of our readers will thank us for pointing out this excellent work to their notice, but they must excuse us for not even attempting an analysis of it. We are deterred from doing so not merely by its bulk, but also by the consideration that it would be alien to the object of our Journal to devote a very large space to the review of a book whose scope has but a distant connexion with the practice of physic. We shall therefore content ourselves with two or three extracts, which may introduce Raspail to our readers: we leave it to them to form an intimate acquaintance with him.

Our author's great instrument of discovery has been the microscope, and he seems to have been led to its use by the following considerations:

"We can understand how, by the help of the crucible and re-agents, the undecomposable elements of an inorganic body may be separated and weighed; and yet how do the difficulties of analysis increase with the number of these elements? But, when we deal with an organized substance, how can we have recourse to the crucible without decomposing the organ? And how shall we employ re-agents to isolate the contained substance, disregarding the obstacle which the organized coats of the organ present to the reaction? Accordingly, from the instant I began to read the works on Organic Chemistry, I could not help a feeling of vagueness; and, notwithstanding the confidence with which the details were given, I all along suspected that the results obtained did not represent the natural state of things. Operations of a kind little employed by the scientific world at last furnished me with the means of explaining the cause of my hesitation, and changing my suspicions into certainty. I saw and delineated organs of excessive minuteness, and whose varied aspects and forms seemed to me to indicate different functions and properties. These organs occur side by side with each other. The eye can distinguish them, but the scalpel cannot separate them. Now, said I to myself, when the chemist bruises, triturates, macerates, or boils in a menstruum, even the smallest fragment of animal or vegetable matter, he must necessarily confound and mix in the same liquid a multitude of substances which nature had placed in separate organs. It would seem as if, confident of the power of his art, he sought to blend every thing together, in order that he might have the pleasure of again separating each part. But, when he has mixed and confounded the whole, he struggles in vain with difficulties which his art has no power to overcome. Hence the contradictions, the incongruities, the extravagances, of the theories to which recourse is had to reconcile inexplicable results; hence the number of undetermined or misnamed substances, of identical bodies viewed as being different, and of new names, which are at last so multiplied as to overload the most attentive memory, and give distaste to the least scrupulous mind.

"It is evident that, if the chemist had understood that each of these substances which he had so unhappily blended, was, by the laws of nature, deposited in a different organ, he would of himself have overturned the scaffolding which he has reared, and done ample justice upon his undecisive results. But, during the whole of the most brilliant period of this mode of chemical observation, this idea never came into any one's mind. The chemist would have spurned at being required to become an anatomist, or a botanist, or a zoologist. He held on his own way, as the botanist and the physiologist did theirs, just like two travellers journeying side by side on two roads, which, in the end, must lead them away from their common object, talking, in the meanwhile, of other people's matters, without seeking to give or receive information respecting their route." (P. xiii.)

The following extracts may serve to show M. Raspail's acuteness as a microscopic observer, as well as his tact in applying his scientific acquirements to the business of real life.

"*Physical Characters of the Granular Particles of this Substance* [*Starch*] *in general.*

"4. This powder, when examined by the microscope, shows nothing but rounded unconnected grains, of variable form and dimensions, not only in different vegetables, but even in the same vegetable. A sufficiently exact notion of these varieties may be formed from the figures delineated in Pl. II. Thus, the fecula of the potatoe (fig. 1.) exhibits grains whose size varies between .0049 and .00013 of an inch, and whose forms are very various, while the largest grains of the fecula of the millet scarcely exceed the latter dimension, and they are all of one form, as far as the limits of the magnifying powers used enable us to judge.

"5. These grains increase in size with the age of the vegetable and of the organ which contains them. In the pericarpium of the ovary of the gramina, previous to fecundation, the grains are not larger than .00013 of an inch; but, after fecundation, they grow until they attain the size of .002 of an inch (Pl. II. fig. 12.)

"6. In certain other organs they change their form at the same time that they increase in size. Thus, in the tubercles of the *Iris florentina* or *germanica*, they possess in the month of June the form and apparent size shown in fig. 13. If the tubercles be left in the open air, and in rather a dark place, they will be found at the end of a fortnight with the singular forms represented in fig. 14., and possessing a size which they scarcely attain till autumn, if left to vegetate in the earth.

"7. There are some vegetables, in the various organs of which the feculent grains are different both in size and form. In the grains of the *Chara hispida* they possess the form and apparent magnitude shown in fig. 3.; but, in the articulations of the same plant, their form and apparent size are those seen in fig. 4. I shall notice all these varieties of form, after treating of the organization of fecula."

" *Refractive Power of the Grains of Fecula, and Appearances connected with it.*

" 8. The shades which are observed on examining the grains of fecula vary according to the kind and the magnifying power of the microscope used.

" 9. If a grain of the fecula of the potatoe be examined, when dry, by transmitted light, its refracting power being considerably different from that of the surrounding air, it will necessarily happen that those rays of the light thrown upon this irregular sphere which impinge obliquely on its inferior surface will be considerably refracted, both when they enter it and when they pass out again, so that scarcely any light will reach the focus of the microscope, except what has traversed the centre of the sphere; consequently, it will appear to the eye of the observer like a black ball having a brilliant round spot in the centre (Pl. II. fig. 21.), or like an irregular black pearl with a somewhat elliptical luminous hole pierced through it (fig. 22.) A bubble of air examined by the microscope under water (Pl. III. fig. 11. *f'* and Pl. VI. fig. 12. *a'*) gives an exactly similar appearance, arising from the same laws of refraction.

" 10. If, on the other hand, the grain of fecula of the potatoe be placed in water (Pl. II. fig. 1.), as its refractive powers differ little from that of the surrounding liquid, it will then exhibit the appearance of a brilliantly-shining pearl; and, if the microscope be as powerfully illuminated as simple microscopes generally are, the transparence of the grain of fecula may be such that it shall be undistinguishable, except by its outline (Pl. II. fig. 23). In this case the grain appears of a larger size, an illusion which is to be corrected by measuring it.

" 11. It is however possible gradually to diminish the transparence of the grain of fecula observed under water, by diminishing the diameter of the cone of light which is thrown by the mirror on its inferior surface. For this purpose a metallic diaphragm is employed, which is pierced with holes of different diameters. We may thus diminish the transparence of the object to such a degree, that the grain of fecula seen under water shall present almost the same appearance which it does when observed in the air. The reason of this is, that by means of this diaphragm the number of rays impinging perpendicularly on the inferior surface of the grain of fecula is diminished, while a greater number are permitted to fall on it obliquely which do not reach the focus of the microscope.

" 12. But if, then, the object-holder be brought nearer, so that the centre of the grain is no longer in the focus of the microscope, the contrary effect will take place. The centre of the grain will appear like a black point in the middle of an illuminated zone, or like an opaque kernel inclosed in a transparent shell; and, if the largest opening of the diaphragm be used in the observation without altering the place of the object-holder, in place of a black point, a luminous spot of a blue colour will be seen." (P. 78.)

" *Fecula of the Æsculus Hippocastanum.* The grains of this fecula vary in their size, according to the size and age of the chesnut. They are very irregular, constricted in the middle of their length like the coccoon of the silkworm, kidney-shaped, or pyriform. The largest does not exceed .0012 of an inch in size. The fruit of this tree, though rich in fecula, and abundant, is not applied to any use, on account of the presence of a bitter matter and a large quantity of potash, which alter the quality of the fecula, and render it unfit for food. Yet by very simple processes it would be easy to convert it to use, and to obtain from it 30 per cent. of fecula, while the potatoe does not yield more than 22 per cent. It would only be necessary to rasp the fruit, as is done with potatoes (94.), and to wash it with water very slightly (23.) acidulated with sulphuric acid, and then to wash out the excess of acid by plenty of water, by which means the fecula would be deprived of every thing that renders it disagreeable and hurtful. Or the process of the starch-maker (104.) might be employed, fermentation being caused by the addition of gluten, or any other fermentable substance.

" Vergnaud recommends the pulp extracted from the horse chesnut, and made into starch, without any other preparation, as an excellent batter for weavers, on account of the deliquescent salt which it contains, and which would allow of their working fine goods in less damp and unwholesome places than they are at present obliged to use, to prevent the batter from drying." (P. 119.)

" For some years past, I have seldom met with wheat in the market of Paris which did not contain an appreciable quantity of the fecula of the potatoe, whose price is so low that the vender may gain 25 per cent. by the mixture. Although its presence does not alter the appearance of the flour, yet, with a little practice, it may be discovered by the unassisted eye, when there is a large proportion of it present. The flour has a crystalline appearance which does not belong to it. By the microscope the fraud is easily detected, and I would undertake to discover it though it were only $\frac{1}{100}$ of the flour. Those who provide bread for public establishments, obliged to purchase the indulgence or the connivance of a great many persons employed in them, adulterate the flour contracted for with farina obtained from coarser substances, such as beans, damaged pease, and even vetches, &c. If a small quantity of this flour can be obtained, or if any of those knots of unbaked farina which the bakers call ' browns' can be found in the bread (76.), it is possible, by the help of the numbers and figures which I have given (93. 119.) to discover the nature of the mixture. No one could fall into any mistake about the fecula of rye, lentils, and potatoes. But, if it should be difficult to ascertain the precise nature of the foreign substance, it will always be easy to obtain a negative result. If a sample of flour be given as wheat flour, and the grains of fecula, instead of being .002 of an inch in size, are mostly under .001, it is evident that the statement is false. To obtain a more positive result, it is necessary to take into account statistical and commercial considerations relative to the price and the place of growth of those substances whose presence is suspected, to resort to analysis on the large scale, and sometimes to the microscopic and comparative analysis of the different organs which are met with in the flour. I shall afterwards speak of the various organs which enable us to recognise by the microscope the farina of the *Cereales*." (P. 131.)

Perhaps one of the most successful parts of M. Raspail's system is the attempt to show that many of the proximate elements of organic bodies are, in fact, mixtures of some other well-known substances: thus, that "tannin is merely an association of resinous matters with an acid (generally the gallic,) which renders them soluble in water (1128.) For, 1st, it has hitherto been found impossible to obtain tannin free from acid; 2d, by treating any resin, or charcoal, with nitric acid, or by acting on camphor, the resins, or some of the gum resins, by sulphuric acid, an artificial tannin may be obtained, possessing all the properties of the tannin of gallnuts or oak-bark; and, 3d, the presence of the acid employed in its preparation may always be detected in artificial tannin, as long as it retains its characteristic property." (P. 491.)

In conclusion, we must remind our readers, especially our younger ones, that this work is not one to be skimmed, or lounged over, or merely read; but that it requires to be studied and worked through, with a microscope and test-tubes ever at hand, as its proper interpreters and commentators. Those who are deterred by the regal price of a fashionable microscope, will be glad to hear that a really useful instrument is by no means expensive: indeed, M. Raspail informs us that Deleuil, of 24, Rue Dauphine, has entered into his enthusiasm for young students, and sells for thirty francs a microscope which could not be had elsewhere for less than eighty.

Dr. Henderson's translation is well executed, and he has enriched it with some useful notes.

